# Elucidating the Impact of Gamma Irradiation Treatment Prior to Aging on Light-Flavor Tartary Buckwheat Baijiu Flavor Profiles: A Multimodal Analysis Combining E-Nose, E-Tongue and HS-GC-IMS

**DOI:** 10.3390/foods15081441

**Published:** 2026-04-21

**Authors:** Zhiqiang Shi, Qing Li, Chen Xia, Yan Wan, Kun Hu, Zhiming Hu, Shengnan Zhong, Yuhan Yang, Yongqing Zhu, Peng Wei, Ke Li

**Affiliations:** 1 Biotechnology and Nuclear Technology Research Institute, Sichuan Academy of Agricultural Sciences, Chengdu 610061, China; zhiqiangshi24@scsaas.cn (Z.S.); lqcd668@scsaas.cn (Q.L.); 13730852451@163.com (S.Z.); mondaytea@163.com (P.W.); 2 Institute of Agro-Products Processing Science and Technology (Institute of Food Nutrition and Health), Sichuan Academy of Agricultural Sciences, Chengdu 610066, China; gnspxiachen@scsaas.cn (C.X.); yuhany@scsaas.cn (Y.Y.); zhuyongqing68@sina.com (Y.Z.); 3 Key Laboratory of Coarse Cereal Processing, Ministry of Agriculture and Rural Affairs, Sichuan Engineering & Technology Research Center of Coarse Cereal Industrialization, School of Food and Biological Engineering, Chengdu University, Chengdu 610106, China; yanwan@cdu.edu.cn; 4 Sinograin Chengdu Depot Co., Ltd., Chengdu 611434, China; kunhu@sicau.edu.cn; 5 Sichuan Institute of Food Inspection, Chengdu 610097, China; huzhiming1126@sina.com

**Keywords:** light-flavor Baijiu, volatile organic compounds, multivariate statistical analysis, odor markers

## Abstract

This study comprehensively analyzed the effects of gamma irradiation (GI) on the flavor profile of aged light-flavor tartary buckwheat Baijiu (LTB) using E-nose, E-tongue, and high-sensitivity headspace–gas chromatography–ion mobility spectrometry (HS-GC-IMS). A total of 30 volatile organic compounds (VOCs) were identified, with concentrations showing significant dose-dependent correlations with GI treatment. Aging alone reduced harsh and pungent VOCs (e.g., 1-propanol, 2-methyl butanoic acid ethyl ester), while GI followed by aging further decreased undesirable compounds (e.g., butanal-D, pyrrolidine) and enhanced beneficial flavor components, such as 1,1-diethoxy ethane-D and butanoic acid propyl ester. Notably, this treatment partially restored 1-propanol, triethylamine, and 2-butanone-M, though their levels remained significantly lower than in newly brewed LTB, achieving a more balanced purity and flavor complexity. The significantly elevated levels of tetrahydrofuran-M/D, 1,1-diethoxy ethane-D, and cyclohexane in GI-treated aged LTB, along with their dose-dependent accumulation patterns, suggest their potential as reliable markers. Multivariate analysis confirmed that all three techniques (E-nose, E-tongue, and HS-GC-IMS) effectively differentiated LTB samples, with strong correlations between E-nose and HS-GC-IMS data, as well as between E-tongue and HS-GC-IMS results. This work provides flavor fingerprints and potential markers for gamma-irradiated LTB identification, while proposing an innovative technical approach for rapid flavor assessment of light-flavor Baijiu.

## 1. Introduction

Chinese Baijiu, a widely favored alcoholic beverage, comprises various styles. Among these, light-flavor Baijiu is particularly popular for its delicate fragrance and smooth taste [1]. Tartary buckwheat (*Fagopyrum tataricum*), a nutritious grain abundant in bioactive compounds, is increasingly regarded as a premium raw material for brewing this distinctive Baijiu [2]. The production of light-flavor Baijiu involves multiple stages, with the aging process being especially critical for harmonizing and refining the final product’s flavor and aromatic profile [3]. Aging typically requires 3~5 years, during which a series of physicochemical reactions reduce the spirit’s harshness and gradually establish an equilibrium between acids and esters, ultimately optimizing its flavor profile [4]. However, prolonged aging increases labor and energy costs for distilleries while extending production cycles. Therefore, to reduce time and costs, researchers have developed methods to accelerate the aging of Baijiu, primarily including chemical-induced aging, physical aging, and biological aging [5]. Among these, physical aging has emerged as the most widely adopted approach due to its operational simplicity and minimal associated pollution [6].

Gamma irradiation (GI), a non-thermal physical processing method, has been demonstrated to enhance the quality of alcoholic beverages [7]. Previous studies have shown that GI can effectively eliminate undesirable flavors and promote the synthesis of flavor-active compounds [8]. The underlying mechanism involves radiation-induced ionization and excitation processes, which generate abundant free radicals and highly reactive molecular species [9]. These activated intermediates subsequently facilitate redox and esterification reactions, thereby accelerating the chemical transformations critical to flavor development during Baijiu aging. The effects of GI on Baijiu are predominantly dose-dependent. For instance, He et al. [10] demonstrated that Baijiu treated with GI at 3 kGy dosage could achieve flavor characteristics equivalent to 1.98 years of natural aging. Notably, the ethyl linoleate content became undetectable at doses ≥50 kGy. These findings underscore the importance of irradiation dosage to modulate VOC profiles for Baijiu quality enhancement. Currently, no studies have explored the effects of varying doses of GI applied prior to aging on the flavor profile of light-flavor tartary buckwheat Baijiu (LTB), particularly in identifying corresponding chemical markers.

The flavor quality of Baijiu is traditionally assessed through human sensory evaluation, an approach that exhibits significant subjectivity [11]. Currently, electronic sensory technologies such as E-nose and E-tongue are gaining popularity in flavor characterization due to their advantages of rapidness, objectivity, and high analytical efficiency [12]. E-tongue enables digital quantification of taste profiles, delivering more sensitive and objective analytical results than human sensory evaluation [13]. While an E-nose can distinguish subtle differences in odor profiles among different samples, it cannot quantitatively analyze individual VOCs [14]. In recent years, high-sensitivity headspace–gas chromatography–ion mobility spectrometry (HS-GC-IMS) has been widely applied in the analysis of VOCs in Baijiu [15,16]. HS-GC-IMS relies on the electrical migration of ionized trace compounds and offers advantages including high sensitivity, rapid analysis, simple operation, and strong visualization capabilities [17]. Therefore, the integration of these three analytical techniques enables a more comprehensive and reliable characterization of the flavor profile in LTB.

This study employed E-nose, E-tongue, and HS-GC-IMS to investigate the effects of GI treatment prior to aging on the flavor profile of LTB. Newly brewed LTB (NL), naturally aged 3-year LTB (0 kGy-AL), and gamma-irradiated then aged 3-year LTB (GI-AL) were comparatively analyzed (for ease of subsequent description, we collectively refer to all LTB that have undergone the three-year aging process as AL, which includes both 0 kGy-AL and GI-AL). Key VOCs were identified through multivariate statistical analysis, and the evaluation of aromatic and taste characteristics was performed using E-nose and E-tongue techniques, supplemented by correlation analysis between E-nose and HS-GC-IMS results, as well as between the results of the E-tongue and HS-GC-IMS. The study aims to clarify the influence of GI treatment at varying doses, applied prior to aging, on the VOC profiles and flavor characteristics of LTB. Furthermore, it seeks to identify specific markers of GI treatment and to determine the key VOCs that differentiate between distinct LTB samples. These findings provide both a theoretical foundation and technical support for detecting gamma-irradiated LTB and improving product quality.

## 2. Materials and Methods

### 2.1. Sample Preparation and GI

High-quality tartary buckwheat grains, provided by the Key Laboratory of Coarse Cereal Processing (Ministry of Agriculture and Rural Affairs) at Chengdu University, were selected to brew tartary buckwheat Baijiu using a standard light-flavor Xiaoqu Baijiu process. The irradiation treatment was performed at the Biotechnology and Nuclear Technology Research Institute of the Sichuan Academy of Agricultural Sciences. A static ^60^Co gamma-ray radiation source with a total initial activity of 1.33 × 10^15^ Bq (35,946 Ci) was used to administer doses of control (0 dose), 2, 4, 6, and 8 kGy at a consistent dose rate of 16.77 Gy/min. Following irradiation, the Baijiu samples were sealed in traditional Baijiu storage ceramic jars and stored in a dedicated Baijiu storage cellar under dark conditions at 25 ± 1 °C for three years. These aged samples were then analyzed alongside freshly brewed tartary buckwheat Baijiu for flavor characteristics.

### 2.2. E-Nose Analysis

The measurements were carried out following the method established by Xia et al. [18], with appropriate modifications. E-nose analysis was performed using a PEN3 electronic nose (Airsense Analytics, Dortmund, Germany), which is equipped with an array of 10 metal oxide semiconductor sensors, each sensitive to different classes of volatile compounds (sensor details are provided in Table 1). For measurement, a 2 mL aliquot of each sample was transferred to a 20 mL headspace vial. The vial was sealed and incubated at room temperature for 30 min to allow for headspace equilibrium. The analysis was then conducted by inserting a probe into the vial headspace. Volatiles were drawn into the sensor chamber at a constant flow rate of 400 mL/min for a measurement time of 60 s.

### 2.3. E-Tongue Analysis

The analytical procedure was based on the method reported by Zhang et al. [19], with slight modifications. Taste analysis was performed using an α-ASTREE electronic tongue (Alpha MOS, Toulouse, France) equipped with seven cross-selective liquid sensors (AHS, ANS, CPS, CTS, NMS, PKS, and SCS). For each measurement, a 25 mL sample aliquot was analyzed for 120 s. The sensor response signals recorded between 101 and 120 s at a sampling frequency of 1 Hz were used for data analysis. To prevent cross-contamination, the sensors were rinsed with ultrapure water for 10 s between each sample measurement.

### 2.4. HS-GC-IMS Analysis

VOCs were analyzed using HS-GC-IMS (FlavourSpec^®^, G.A.S., Dortmund, Germany) on the basis of a previous study [20]. A 4 mL sample was diluted 20-fold, and a 4 mL aliquot of this dilution was transferred to a 20 mL headspace vial. The vial was sealed and incubated at 50 °C for 15 min with an agitation speed of 500 rpm. A 100 µL aliquot of the headspace was automatically injected into the GC-IMS. Separation was performed on a MXT-5 metal capillary column (15 m × 0.53 mm, 1 µm) maintained at 60 °C. High-purity nitrogen (99.999%) was used as both the carrier and drift gas. The drift gas flow was maintained at a constant 150 mL/min, and the IMS detector temperature was set to 45 °C. Compound identification was performed by comparing the GC retention index and IMS drift time to standards in the NIST and IMS databases using the instrument’s Library Search software (VOCal (0.4.03)). A semi-quantitative approach, based on the peak volume of the compound, was used for quantification.

### 2.5. Statistical Analysis

All experiments were performed in triplicate, and data are presented as mean ± standard deviation (SD). Multivariate statistical analyses, including principal component analysis (PCA), cluster analysis, and partial least squares-discriminant analysis (PLS-DA), were conducted using SIMCA 14.1 software. The data significance analysis was performed using WPS software (2023). The correlation heatmap was drawn using ChiPlot (2025) (https://www.chiplot.online/) (accessed on 3 July 2025).

## 3. Results and Discussion

### 3.1. Evaluation by E-Nose

**Figure 1:**
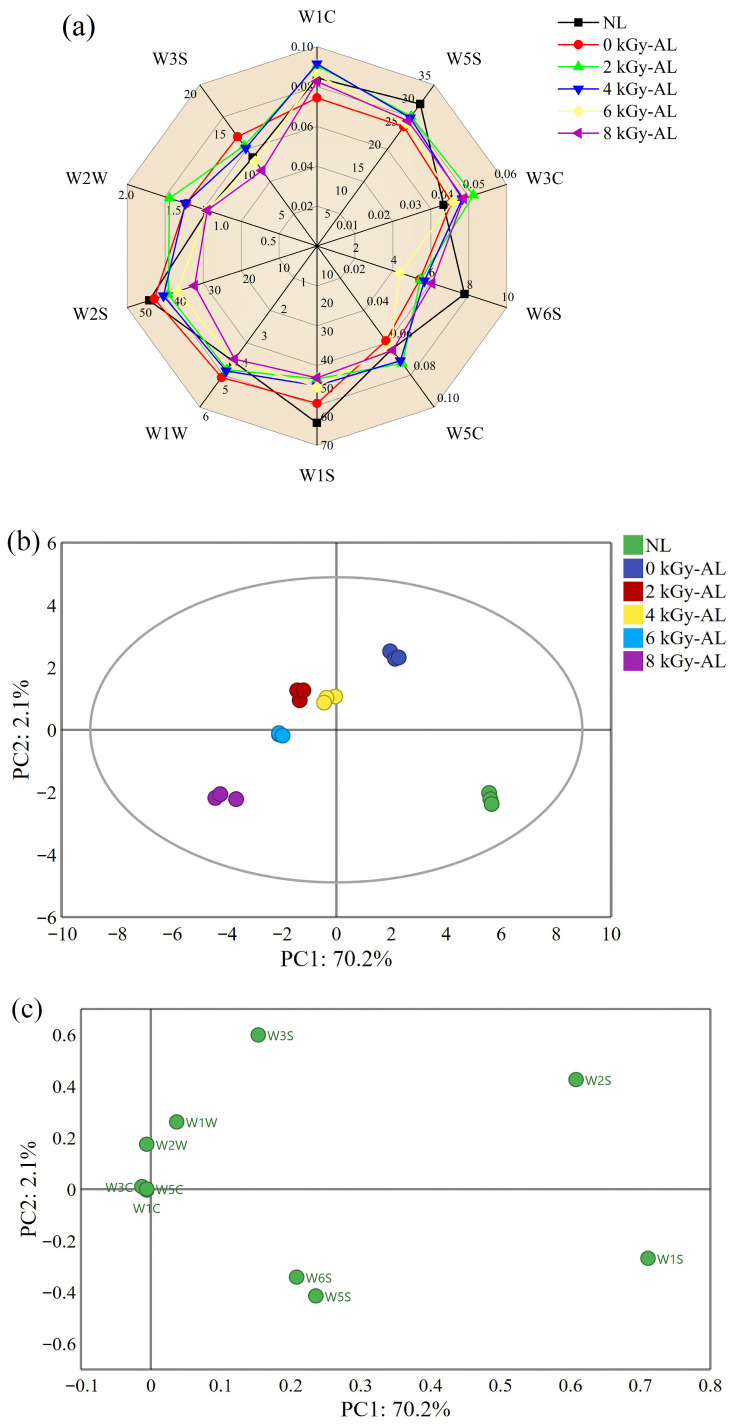
(**a**) Radar chart of E-nose response data; (**b**) principal component analysis (PCA) score plot of E-nose response data; (**c**) PCA loading plot of E-nose response data. NL: newly brewed light-flavor tartary buckwheat baijiu; 0 kGy-AL: naturally aged light-flavor tartary buckwheat baijiu for 3 years; 2/4/6/8 kGy-AL: light-flavor tartary buckwheat baijiu that underwent gamma irradiation at doses of 2, 4, 6, and 8 kGy followed by a 3-year aging period.

The E-nose provides a relatively straightforward analytical technique for objective VOC identification. Figure 1a presents the E-nose response profiles of different LTB samples. The W1S sensor demonstrated the highest response intensity, followed sequentially by W2S, W5S, and W3S sensors, indicating that they could be identified as feature sensors. Notably, NL exhibited higher W1S, W2S, W5S, and W6S sensor values than AL, indicating a greater abundance of methyl compounds, nitrogen oxides, hydrides, alcohols, aldehydes, and ketones in NL. Comparative analysis revealed that 0 kGy-AL samples showed elevated responses in W1S, W1W, W2S, and W3S sensors relative to GI-AL samples, suggesting higher concentrations of methyl compounds, sulfides, pyrazines, terpenes, alcohols, aldehydes, and ketones in non-irradiated LTB. Interestingly, GI-AL samples displayed stronger W3C sensor responses, reflecting their richer aromatic compound content.

PCA, a multivariate statistical method for dimensionality reduction through principal component extraction, was employed to evaluate overall differences. As shown in Figure 1b, the first two principal components (PC1 and PC2) accounted for 70.2% and 2.1% of total variance, respectively, demonstrating PCA’s effectiveness in explaining LTB variance [21]. The analysis revealed clear separation among LTBs with different irradiation doses, indicating distinct flavor profiles. Specifically, significant separation between GI-AL and both NL and 0 kGy-AL samples, confirming that GI treatment substantially alters volatile flavor profiles. Notably, 2 kGy-AL and 4 kGy-AL clustered closely within the fourth quadrant, indicating shared flavor characteristics. While 6 kGy-AL showed proximity to 2 kGy-AL, their distinct quadrant positioning revealed flavor differences. Importantly, 6 kGy-AL and 8 kGy-AL co-localized in the third quadrant, suggesting similar flavor patterns between these irradiation treatments. Analysis of sensor loadings (Figure 1c) demonstrated that the flavor distinction between 0 kGy-AL and GI-AL was primarily associated with responses from W3S, W1C, W5C, W3C, W2W, and W1W sensors. Specifically, W1C, W5C, and W3C showed negative correlations with PC1, while W3S and W1W exhibited a strong positive correlation. In contrast, W3S, W1W, and W2W were strongly positively correlated with PC2, whereas W1C, W5C, and W3C showed negligible correlation. Based on these patterns combined with sensor performance characteristics, we identified aromatic compounds, alkanes, terpenes, and sulfur-containing compounds as key odor markers. While the E-nose effectively characterized global odor patterns across LTB samples, its inability to provide detailed VOC speciation necessitated subsequent HS-GC-IMS analysis for comprehensive volatile compound identification.

### 3.2. Evaluation by E-Tongue

**Figure 2:**
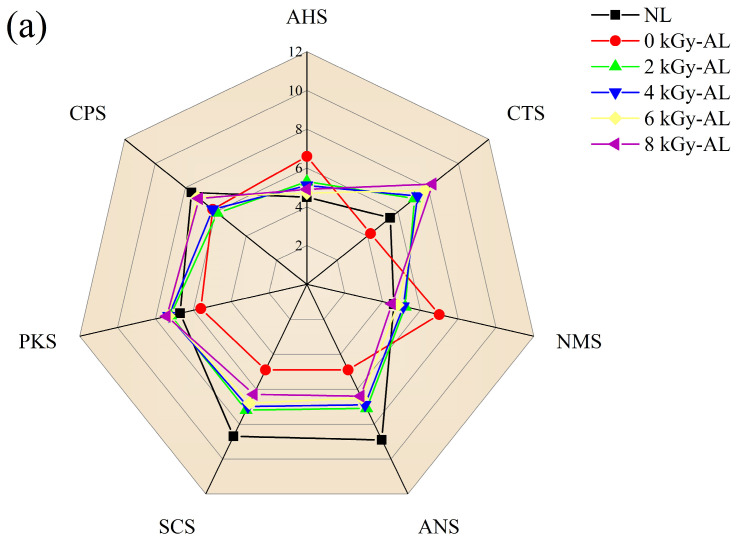

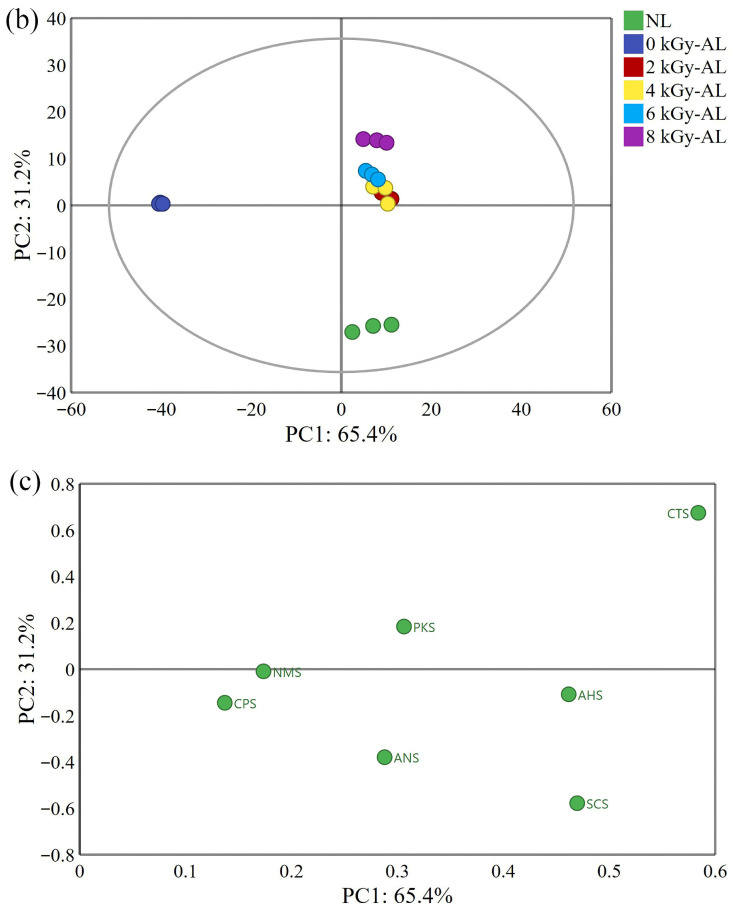
(**a**) Radar chart of E-tongue response data; (**b**) principal component analysis (PCA) score plot of E-tongue response data; (**c**) PCA loading plot of E-tongue response data. NL: newly brewed light-flavor tartary buckwheat baijiu; 0 kGy-AL: naturally aged light-flavor tartary buckwheat baijiu for 3 years; 2/4/6/8 kGy-AL: light-flavor tartary buckwheat baijiu that underwent gamma irradiation at doses of 2, 4, 6, and 8 kGy followed by a 3-year aging period.

The E-tongue offers significant advantages for objectively, simply, and rapidly characterizing the taste profiles of Baijiu. In this study, seven specialized sensors were employed to evaluate taste characteristics, including five taste-specific sensors for sourness (AHS), saltiness (CTS), umami (NMS), bitterness (SCS), and sweetness (ANS), along with two general-purpose sensors [18]. As illustrated in Figure 2a, comparative analysis revealed that NL exhibited more pronounced bitter and sweet taste characteristics, while the 0 kGy-AL displayed stronger sour, salty, and umami attributes. Notably, the GI-AL exhibited a pronounced saltiness profile. Moreover, compared to the 0 kGy-AL group, it also demonstrated enhanced sweetness and bitterness intensities.

The PCA score plot derived from E-tongue data (Figure 2b) demonstrated effective discrimination, with PC1 and PC2 accounting for 65.4% and 31.2% of total variance, respectively, collectively capturing the major taste characteristics. The PCA results showed significant separation between NL and both 0 kGy-AL and GI-AL along PC1 and PC2, respectively. These results demonstrate that both natural aging and GI treatment followed by aging can induce substantial taste modifications. Furthermore, the analysis indicates notable taste differences between GI-AL and 0 kGy-AL samples. Notably, the taste difference between 0 kGy-AL and NL was more pronounced than that between GI-AL and NL, suggesting that radiation-induced free radical reactions may promote premature decomposition or transformation of certain taste precursors, thereby reducing their subsequent evolution during the aging process. Furthermore, all irradiated samples (2/4/6/8 kGy-AL) clustered closely in the first quadrant, indicating minimal differences in taste characteristics across different irradiation doses and suggesting that the irradiation dose has a limited influence on the final taste profile of LTB. Sensor loadings analysis (Figure 2c) revealed that the flavor differences between 0 kGy-AL and GI-AL were associated with responses from all taste sensors, indicating substantial divergence in their taste profiles. In contrast, the differences between NL and GI-AL groups correlated with all sensors except NMS, consistent with the minimal NMS response variation observed in Figure 2a. Among these sensors, CTS and PKS showed positive correlations with PC2, whereas CPS, ANS, AHS, and SCS exhibited negative correlations with PC2, aligning with the pronounced saltiness in GI-AL and the dominant sweetness and bitterness in NL. Although E-tongue effectively characterized the overall taste profile of LTB samples, identifying VOCs contributing to these distinct taste attributes requires further HS-GC-IMS analysis and correlation studies.

### 3.3. Analysis of VOCs by HS-GC-IMS

#### 3.3.1. The Overall Differences in VOCs

**Figure 3:**
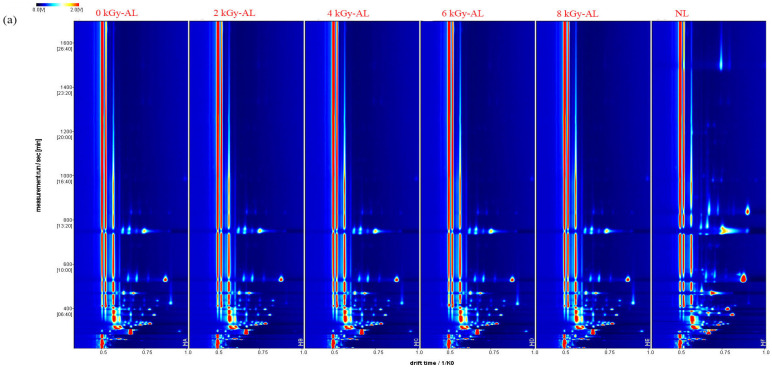

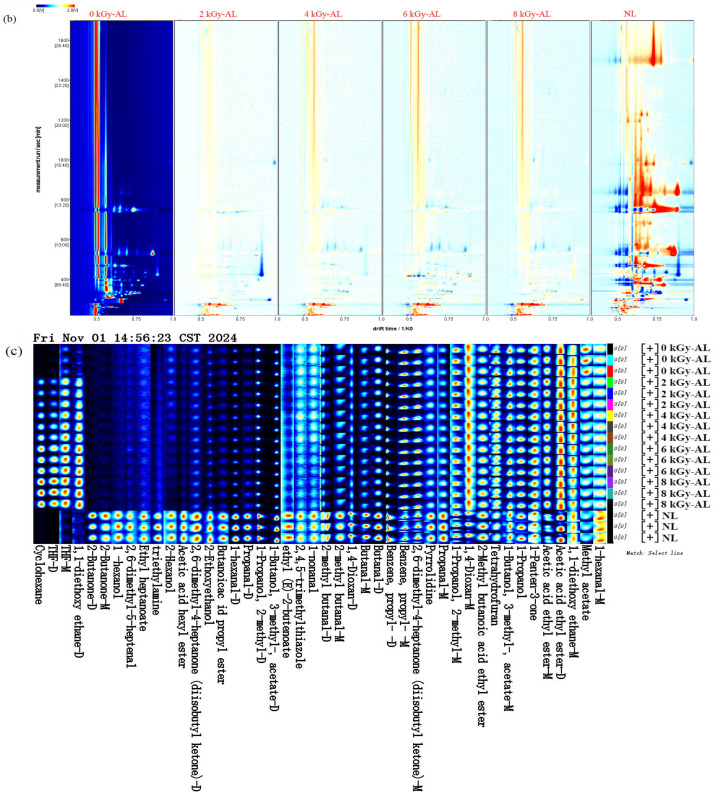
HS-GC-IMS volatile compound analysis in differently treated light-flavor tartary buckwheat baijiu: (**a**) two-dimensional topographic plots of volatile compounds; (**b**) NL and 2/4/6/8 kGy-AL by using 0 kGy-AL as a reference and the comparative analysis topographic plots; (**c**) fingerprints of volatile compounds. NL: newly brewed light-flavor tartary buckwheat baijiu; 0 kGy-AL: naturally aged light-flavor tartary buckwheat baijiu for 3 years; 2/4/6/8 kGy-AL: light-flavor tartary buckwheat baijiu that underwent GI at doses of 2, 4, 6, and 8 kGy followed by a 3-year aging period.

**Figure 4:**
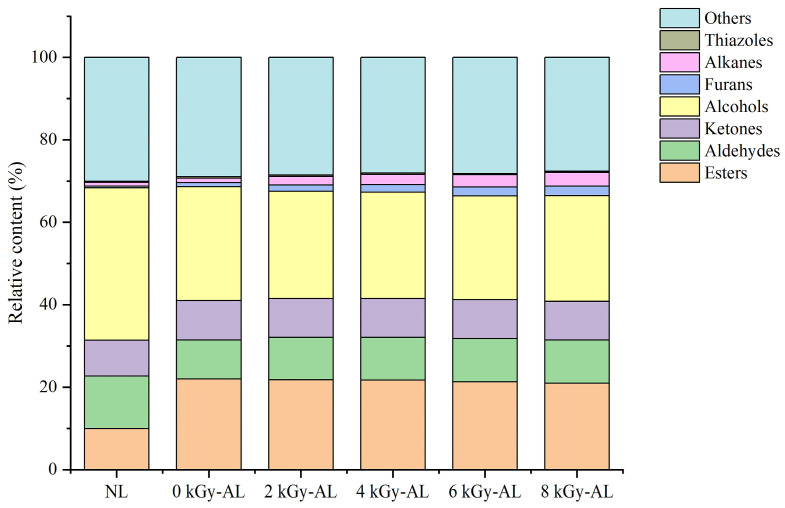
The relative percentage content of volatile components in the differently treated light-flavor tartary buckwheat baijiu, determined using HS-GC-IMS. NL: newly brewed light-flavor tartary buckwheat baijiu; 0 kGy-AL: naturally aged light-flavor tartary buckwheat baijiu for 3 years; 2/4/6/8 kGy-AL: light-flavor tartary buckwheat baijiu that underwent gamma irradiation at doses of 2, 4, 6, and 8 kGy followed by a 3-year aging period.

Although the 0 kGy-AL and GI-AL samples exhibited similar VOC peak patterns (Figure 3a), each sample displayed distinct signal characteristics, indicating significant differences in VOCs. The comparison topographic plots (Figure 3b) effectively delineate VOC variations. Relative to 0 kGy-AL, regions in red indicate increased VOC concentrations, blue denotes decreased concentrations, and white represents comparable levels. As shown, NL exhibited significantly higher VOC diversity and concentrations than 0 kGy-AL. This discrepancy arises because VOCs in NL have not undergone natural volatilization or chemical transformation, which explains their more intense, pungent aroma and potential off-odors. In contrast, aging facilitates the volatilization or conversion of irritant compounds while retaining high-boiling-point flavor substances, resulting in a more harmonious aroma [22]. Compared with 0 kGy-AL, GI-AL exhibited significant alterations in multiple VOC contents across different irradiation doses, a phenomenon attributable to radiation-induced complex chemical reactions. For instance, free radicals generated by GI may cleave small ester molecules and react with other wine components to form larger long-chain esters [23]. To systematically evaluate VOC differences among NL, 0 kGy-AL, and GI-AL samples, we generated characteristic fingerprint spectra (Figure 3c). In these spectra, each row represents the signal peak intensity of different volatile compounds within the same sample, while each column corresponds to the peak intensity across different samples. The color gradient reflects relative VOC abundance, with brighter hues indicating higher peak intensities. As evidenced in Figure 4, GI-AL exhibited significantly higher concentrations of THF-M/D, 1,1-diethoxyethane-D, and cyclohexane compared to both NL and 0 kGy-AL samples. Notably, these compounds demonstrated a clear dose-dependent accumulation pattern with increasing irradiation intensity (2–8 kGy).

#### 3.3.2. HS-GC-IMS Integral Parameter Analysis of VOCs

Qualitative analysis based on retention time, retention index, and drift time identified 44 characteristic signal peaks (Table 2). Due to limitations in database availability, we identified 30 representative compounds (including both monomers and dimers) and characterized their odor properties. These included 7 esters, 5 aldehydes, 6 alcohols, 4 alkanes, 3 ketones, 1 furan, 1 thiazole, and 3 other compounds. Figure 4 clearly demonstrates that aging-induced VOC changes are primarily characterized by an overall increase in ester content coupled with decreases in total alcohol and aldehyde concentrations. Specifically, 0 kGy-AL and 2 kGy-AL showed 120.5% and 118.6% higher ester levels, respectively, compared to NL, while exhibiting 25.3% and 29.6% reductions in alcohol content and 25.7% and 19.7% decreases in aldehyde levels versus NL. In contrast, GI affected VOC profiles through dose-dependent increases in total alkane and furan compounds, as corroborated by fingerprint analysis. The 8 kGy-AL exhibited 266.83% and 183.72% higher total alkane content compared to NL and 0 kGy-AL, respectively. Similarly, the total furan content in 8 kGy-AL rose by 439.20% and 133.57% relative to NL and 0 kGy-AL. The detailed analysis of VOCs is explained below.

Esters, characterized by their low odor thresholds and synergistic effects, serve as the primary aroma-active compounds in Baijiu, imparting fruity, floral, and sweet notes [24]. In light-flavor Baijiu, ethyl acetate dominates the aroma profile, while supplementary esters contribute to its layered complexity [25]. Our analysis indicates that the content of the five esters in AL is significantly lower compared to NL: ethyl heptanoate, butanoic acid propyl ester, acetic acid hexyl ester, ethyl (E)-2-butenoate, and 2-methyl butanoic acid ethyl ester. This reduction may be attributed to hydrolysis of long-chain esters (e.g., ethyl heptanoate, acetic acid hexyl ester) into corresponding acids and alcohols during storage, and oxidation or volatilization of medium/short-chain esters (e.g., ethyl (E)-2-butenoate, butanoic acid propyl ester) [26]. It is noteworthy that the content of butanoic acid propyl ester in GI-AL was significantly higher than in 0 kGy-AL, resulting in improved flavor complexity in GI-AL. Furthermore, this compound exhibited a dose-dependent increase. This phenomenon may be attributed to the radiolysis of water molecules under elevated irradiation doses, which generates reactive free radicals such as H· and ·OH. These active species subsequently accelerate redox reactions and promote esterification processes within the liquor system [27]. According to the description of the odor description, the decreased concentrations of ethyl (E)-2-butenoate and ethyl 2-methylbutanoate attenuated the pungent and sharp aroma in LTB, thereby accentuating the characteristic pure and clean aftertaste typical of light-flavor Baijiu. The 2 kGy-AL showed the lowest levels of the two compounds. Conversely, AL exhibited marked increases in acetic acid ethyl ester-D and methyl acetate. The elevated acetic acid ethyl ester content enhanced the fruity aroma, consistent with Yang et al.’s findings in GI treated light-flavor erguotou Baijiu (8 kGy) [23]. Methyl acetate contributes a fresh note at low concentrations but imparts undesirable solvent-like off-odors at higher levels [28]. Importantly, compared with 0 kGy-AL, GI treatment appropriately reduced the methyl acetate content, preserving the flavor quality of LTB.

Aldehydes, which are primarily derived from alcohol oxidation, represent important aromatic constituents in Chinese Baijiu [29]. The study revealed significant decreases in the concentrations of 2,6-dimethyl-5-heptenal, 1-hexanal-D, 1-nonanal, 2-methyl butanal-M/D, butanal-M/D, and propanal-M/D in AL compared to NL. This reduction could be explained by several factors: aldehydes are easily oxidized to corresponding carboxylic acids, particularly during extended storage or irradiation [30]; they may participate in aldol condensation to form esters; and GI might directly cleave aldehyde molecules (such as through C=O bond breakage) or facilitate their conversion via free radical reactions [25]. The diminished aldehyde content exerts dual effects on the quality of light-flavor Baijiu. On one hand, it helps alleviate undesirable odors since low-threshold aldehydes such as butanal and 1-hexanal, which typically impart green, musty, or fatty off-notes, are reduced, resulting in a purer liquor character. The decreased levels of 2-methyl butanal (with musty/cocoa/nutty notes) and 1-nonanal (waxy character) may shift the aroma profile toward a fresher and crisper style rather than a heavy one. On the other hand, the loss of 2,6-dimethyl-5-heptenal, which contributes melon-like sweetness, could compromise some fruity complexity in the aroma. The butanal-D content showed a dose-dependent decrease with increasing irradiation, likely because higher irradiation doses generated more free radicals in the liquor, thereby accelerating redox and esterification processes [8]. The content of propanal-M/D in AL was significantly lower than in NL, whereas in GI-AL, its levels showed a marked increase compared to 0 kGy-AL, with a clear positive correlation with irradiation dose. This phenomenon may be attributed to the ·OH produced by irradiation, which may oxidize 1-propanol to form propanal. The moderate recovery of propanal-M/D after irradiation helps compensate for the loss of whiskey-like roundness during aging, preventing the liquor body from becoming too thin. More notably, the content of 1,1-diethoxy ethane-D (commonly known as acetal) in GI-AL was substantially higher than in both NL and 0 kGy-AL, showing a positive correlation with irradiation dose. This increase may help offset the loss of ethereal and green notes during aging. Acetal compounds impart distinctive fruity notes (sweet with slight astringency) and synergistically interact with other aroma substances to form the characteristic bouquet of Baijiu [31]. As one of the important indicators of Baijiu aging, acetal content continuously increases with extended storage time [32]. These results collectively demonstrate that GI treatment significantly enhanced the aging degree of LTB.

Ketones represent another important class of flavor compounds in Baijiu, although excessive concentrations can impart undesirable bitter notes [33]. The reduction in ketones may contribute to improved taste quality. Comparative analysis revealed significantly decreased levels of 2-butanone-M/D, 2,6-dimethyl-4-heptanone (diisobutyl ketone)-M/D, and 1-penten-3-one in AL relative to NL. These changes in ketone content may result not only from irradiation effects but also from the combined influence of volatilization, diffusion during storage, and environmental conditions [34]. Notably, GI-AL exhibited substantially lower 2,6-dimethyl-4-heptanone (diisobutyl ketone)-D content compared to 0 kGy-AL, though this reduction did not demonstrate dose dependency. The reduction in ketones aligns with previous findings demonstrating the near-complete disappearance of ketones in Daqu Baijiu following irradiation (≥4 kGy), thereby corroborating the significant impact of irradiation treatment on ketone compounds in distilled spirits [35].

Alcohols serve as the primary flavor components in Baijiu, produced during fermentation through the metabolic conversion of sugars, proteins, and amino acids. These compounds significantly contribute to the Baijiu’s smooth texture and overall quality [36]. Our analysis revealed marked decreases in the concentrations of 2-ethoxyethanol, 1-butanol (3-methyl-,acetate-M and -D forms), 1-propanol (including its 2-methyl-D derivatives), along with 1-hexanol and 2-hexanol in AL compared to NL. The reduction in 2-ethoxyethanol content may help mitigate intoxication risks and decrease the liquor’s irritancy, as high-concentration ethanol exerts direct effects on drinkers’ brain function. 1-propanol, recognized as one of the compounds responsible for astringency in Baijiu, can produce pungent aromas and a sharp, spicy taste when present in excessive amounts [37]. Its decreased concentration consequently contributes to a purer liquor character. Compared to 0 kGy-AL, the 1-propanol content in GI-AL exhibited a dose-dependent increase. This modulation contributed to maintaining desirable flavor complexity while mitigating excessive astringency and pungency in the liquor body. Furthermore, in GI-AL, the contents of 1-butanol (3-methyl-,acetate-M and -D forms) showed further reduction compared to 0 kGy-AL, though without demonstrating dose dependency. This phenomenon may result from irradiation-induced acceleration of esterification reactions between alcohols and acids, or alternatively through free radical-mediated oxidation of alcohol compounds [9]. However, the concurrent decrease in 1-hexanol and 2-hexanol levels might lead to a thinner mouthfeel in the liquor, potentially diminishing its characteristic smoothness and roundness [23].

Furan compounds, typically formed through non-enzymatic browning reactions in Baijiu, generally exhibit characteristic almond and caramel-like aroma profiles. These compounds likely contribute to the formation of the distinctive “cellar aroma” sensory experience by interacting with other aromatic components [38]. Our study revealed significantly higher concentrations of tetrahydrofuran (THF) and its isotopic variants (THF-M and THF-D) in AL compared to NL. Furthermore, GI-AL showed markedly elevated THF levels relative to 0 kGy-AL, with a clear positive correlation with irradiation dose. This phenomenon may be attributed to GI-induced water radiolysis, which produces reactive ·H, which acts as a reducing agent, participating in the reduction of aldehydes/alcohols and subsequently promoting THF formation.

Alkanes are flavor compounds synthesized during the late fermentation stage of light-flavor Baijiu, though they do not constitute the predominant flavor-contributing substances in the liquor [39]. Comparative analysis revealed that cyclohexane levels in 0 kGy-AL showed a slight decrease compared to NL, likely due to the gradual oxidation of cyclohexane during aging. More remarkably, GI treatment induced a substantial, dose-dependent increase in cyclohexane content. Notably, the content of pyrrolidine (imparting ammoniacal, animalic, and egg-like odors) in AL was significantly lower than in NL, with GI treatment causing further reduction. This decrease contributes to a cleaner and fresher flavor profile in LTB. The observed reduction may be attributed to two mechanisms: preferential oxidation of nitrogen-containing heterocyclic compounds by radiation-generated ·OH, and potential radiation-induced degradation of proline, the key biosynthetic precursor of pyrrolidine [40].

In addition to the aforementioned VOCs, we observed significant changes in other VOCs during the aging process. The content of 2,4,5-trimethylthiazole (imparting musty and nutty notes) was markedly lower in AL compared to NL, with the 6 kGy-AL showing the lowest levels, contributing to AL’s cleaner and more refreshing character. Notably, AL also exhibited significantly reduced levels of benzene, propyl-M/D, and 1,4-dioxane-M/D compared to NL, which likely diminishes solvent-like off-odors in the aged product. Similarly, the concentration of triethylamine (associated with ammoniacal and fishy odors) was substantially lower in AL, resulting in purer liquor quality, though with a potential slight reduction in “fermentation character”. Interestingly, GI treatment caused a moderate rebound in triethylamine content while maintaining levels significantly below NL, which may help restore some fermentation complexity and enhance flavor layering without compromising overall purity.

In summary, the aging process of LTB leads to a significant reduction in numerous VOCs associated with pungent and unpleasant odors, which aligns with the characteristic maturation pattern of light-flavor Baijiu. GI treatment further refines the liquor profile, achieving a clean and refreshing character while maintaining desirable flavor complexity. Notably, GI-AL exhibited remarkably elevated levels of THF-M/D, 1,1-diethoxy ethane-D, and cyclohexane compared to 0 kGy-AL, with their concentrations showing a clear positive correlation with irradiation dose. These radiation-specific changes suggest their potential as chemical markers for gamma-irradiated LTB. However, further research is necessary to establish precise dose–response models quantifying their formation kinetics and validating their reliability as irradiation indicators.

#### 3.3.3. PCA and PLS-DA Analysis of VOCs

**Figure 5:**
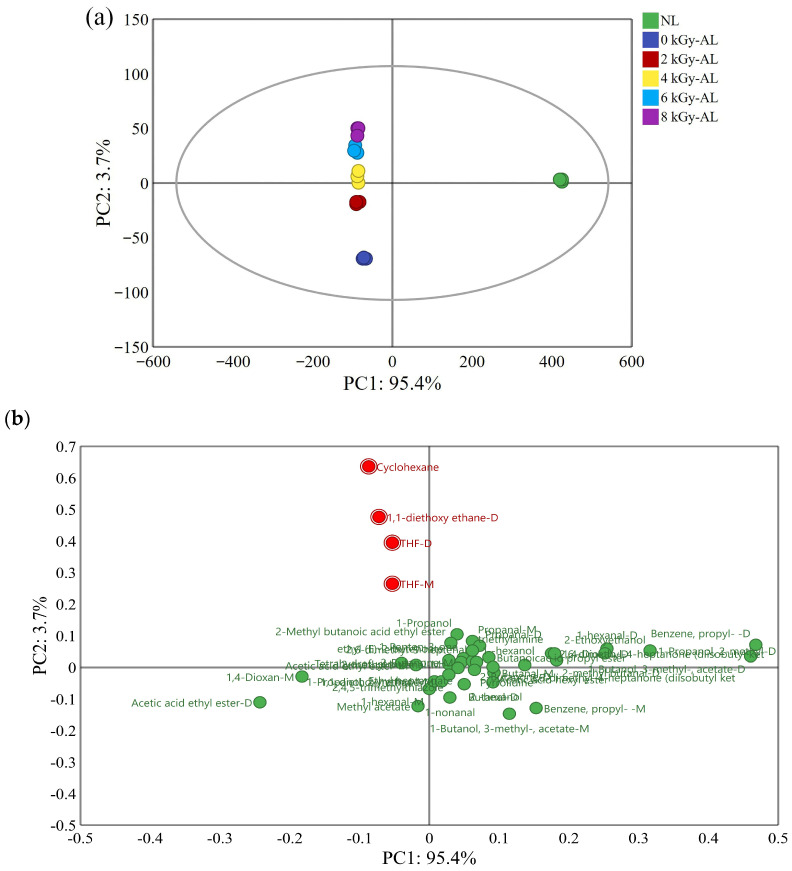
(**a**) Principal component analysis (PCA) score plot based on HS-GC-IMS; (**b**) PCA loadings plot based on HS-GC-IMS. NL: newly brewed light-flavor tartary buckwheat baijiu; 0 kGy-AL: naturally aged light-flavor tartary buckwheat baijiu for 3 years; 2/4/6/8 kGy-AL: light-flavor tartary buckwheat baijiu that underwent gamma irradiation at doses of 2, 4, 6, and 8 kGy followed by a 3-year aging period.

To effectively differentiate the flavor profiles among various LTB samples, multivariate statistical analysis was performed on six experimental groups. We initially performed PCA using the peak signal intensities of VOCs (Figure 5a). The cumulative contribution rate of PC1 and PC2 reached 99.1%, enabling clear classification of the six LTB sample groups based on their aroma characteristics. The PCA results showed that NL clustered on the positive axis of PC1, while AL grouped on the negative axis, indicating that aging significantly modified the VOC profile of LTB. Notably, both 0 kGy-AL and GI-AL were distributed on the negative axis of PC1, demonstrating that GI induced VOC changes qualitatively similar to those occurring during natural aging processes. In addition, significant separation between NL and AL in the PCA score plot, as well as marked distance between 0 kGy-AL and GI-AL, indicates substantial differences between these groups, which is consistent with the E-nose and fingerprint analysis results. The close proximity of 6 kGy-AL and 8 kGy-AL in the PCA score plot, along with their minimal differences in the fingerprint profiles, suggests no major VOC variations exist between these two irradiation doses. Considering energy efficiency, a 6 kGy irradiation dose may be sufficient for LTB treatment. The loading plot in Figure 5b more clearly demonstrates the differences between compounds, with results consistent with the fingerprint analysis in demonstrating that GI-AL contains higher concentrations of specific VOCs, including THF-M/D, 1,1-diethoxy ethane-D, cyclohexane, and acetic acid ethyl ester-D.

**Figure 6:**
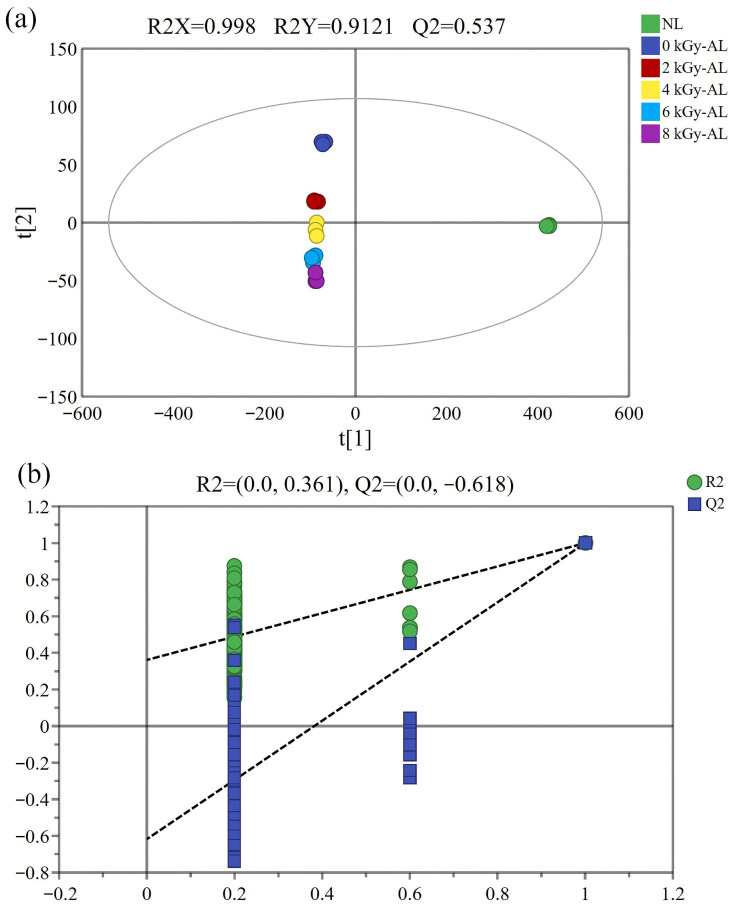

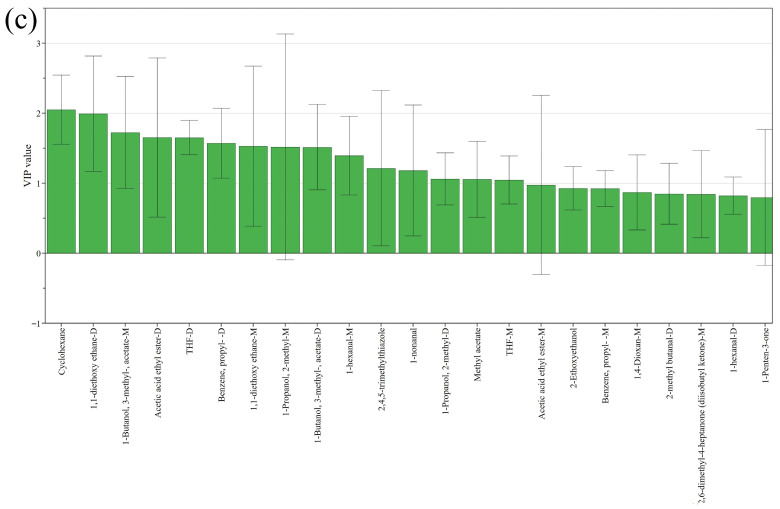
(**a**) Partial least squares-discriminant analysis (PLS-DA) score plot based on HS-GC-IMS; (**b**) PLS-DA permutation tests based on HS-GC-IMS; (**c**) PLS-DA variable importance projection (VIP) values based on HS-GC-IMS. NL: newly brewed light-flavor tartary buckwheat baijiu; 0 kGy-AL: naturally aged light-flavor tartary buckwheat baijiu for 3 years; 2/4/6/8 kGy-AL: light-flavor tartary buckwheat baijiu that underwent gamma irradiation at doses of 2, 4, 6, and 8 kGy followed by a 3-year aging period.

**Figure 7:**
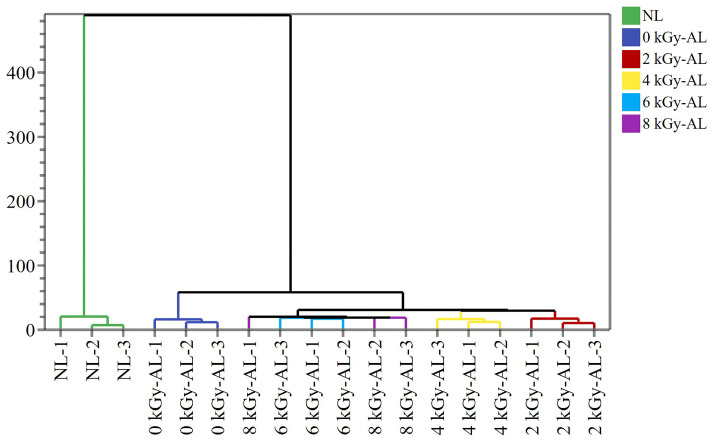
A cluster analysis of the volatile components in differently treated light-flavor tartary buckwheat baijiu. NL: newly brewed light-flavor tartary buckwheat baijiu; 0 kGy-AL: naturally aged light-flavor tartary buckwheat baijiu for 3 years; 2/4/6/8 kGy-AL: light-flavor tartary buckwheat baijiu that underwent gamma irradiation at doses of 2, 4, 6, and 8 kGy followed by a 3-year aging period.

PCA focuses on sample classification, while PLS-DA emphasizes discriminant analysis [41]. As shown in Figure 6a, the PLS-DA model demonstrated excellent quality with three key metrics: R2X = 0.998, R2Y = 0.912, and Q2 = 0.537. Both the explanation rate (R2) and prediction rate (Q2) exceeded 0.5, indicating robust explanatory and predictive capabilities of the model. The six groups showed distinct clustering in the PLS-DA score plot with minimal intra-group variation. There is obvious spatial separation between AL and NL along the PC1. AL clustered on the left side of PC1, while NL was positioned on the far right. Notably, GI-AL showed significant spatial separation from 0 kGy-AL along the PC2. To prevent overfitting, permutation testing with 200 iterations was performed on the established PLS-DA model. As illustrated in Figure 6b, both R2 and Q2 parameters surpassed the permutation test results, and the Q2 scatter plot regression line’s intercept with the vertical axis was below zero, confirming successful model validation and good data-model fit. Subsequently, VIP analysis was conducted to identify the most discriminative VOCs among the six groups [42]. Using VIP > 1 as the threshold, 15 key VOCs were selected (Figure 6c), which effectively differentiated the sample groups based on their irradiation treatments and aging characteristics. Based on the analytical results, we identified discriminant marker components across different treatment groups by applying the single-variable criterion of the one-way ANOVA (*p* < 0.05) and VIP> 1.0 (Figure 6c and Table 2). We found that 1-butanol,3-methyl-,acetate-M/D, acetic acid ethyl ester-D, benzene,propyl-D, 2,4,5-trimethylthiazole, 1-nonanal, 1-propanol,2-methyl-D, methyl acetate, and THF m served as characteristic VOCs distinguishing NL from AL. Further analysis demonstrated that four signature VOCs (cyclohexane, 1,1-diethoxy ethane-D, THF-M/D), along with benzene,propyl-D, and methyl acetate, were the key compounds responsible for flavor differences between 0 kGy-AL and GI-AL. Meanwhile, 1-butanol,3-methyl-,acetate-M, benzene,propyl-D, 1-hexanal-M, 2,4,5-trimethylthiazole, 1-nonanal, and THF m showed significant variations between 2 kGy-AL and 4 kGy-AL. Additionally, the four signature VOCs, along with 2,4,5-trimethylthiazole and 1-nonanal, were identified as discriminative markers for 4 kGy-AL versus 6 kGy-AL. Notably, 6 kGy-AL and 8 kGy-AL groups exhibited similar flavor profiles, with only the four signature VOCs showing significant differences. Cluster analysis results (Figure 7) further validated these findings: NL and AL groups formed distinct, separate clusters; 0 kGy-AL and GI-AL groups belonged to different sub-clusters; 2 kGy-AL and 4 kGy-AL groups each formed independent clusters; while 6 kGy-AL and 8 kGy-AL groups clustered together. This clustering pattern showed strong consistency with VOC analysis results, collectively revealing flavor characteristic differences among treatment groups.

### 3.4. Correlation Analysis of HS-GC–IMS Results with E-Nose and E-Tongue

**Figure 8:**
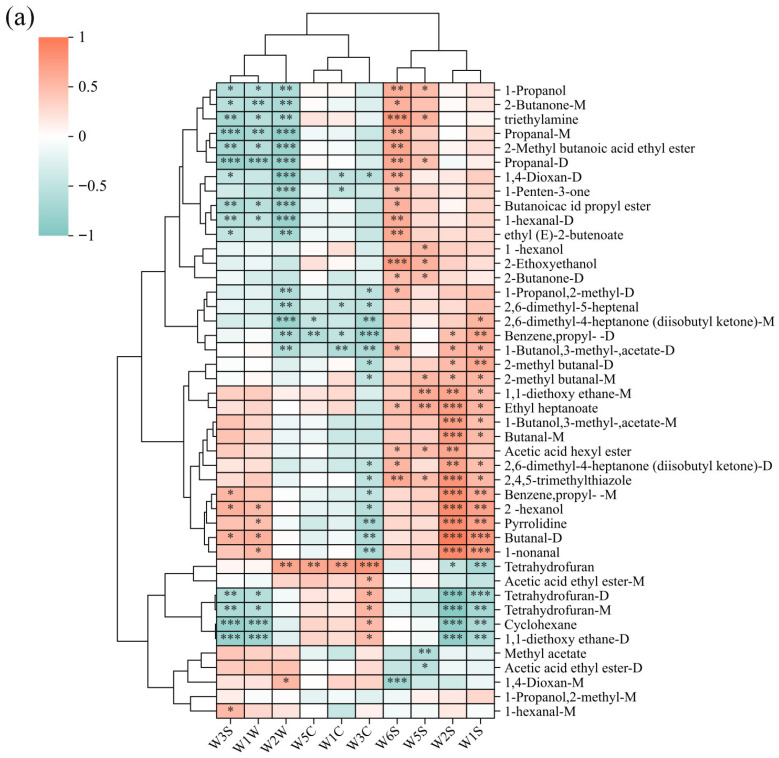

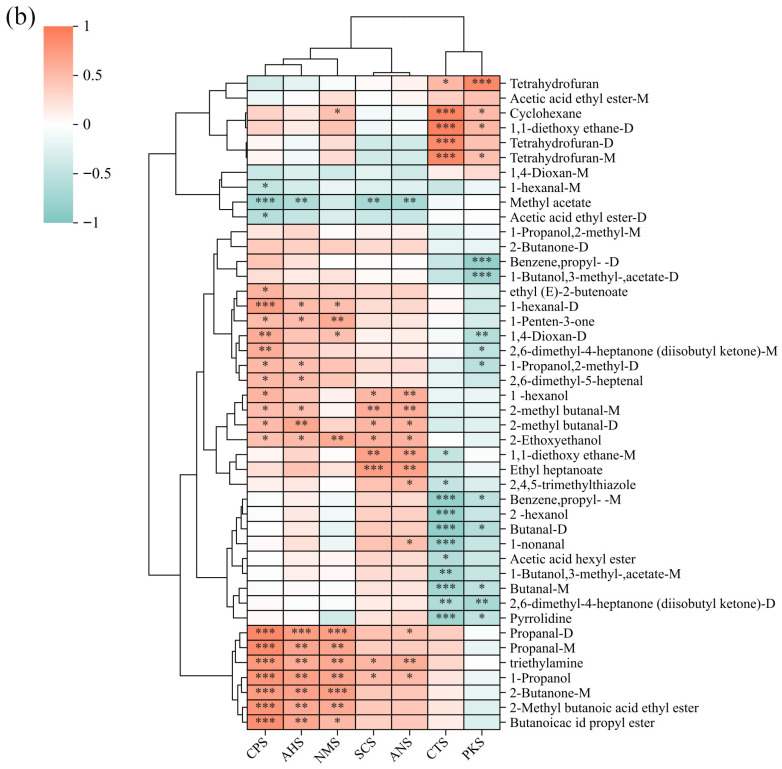
(**a**) Spearman’s correlation heatmap showing the correlation between the volatile compound levels and E-nose sensor; (**b**) Spearman’s correlation heatmap showing the correlation between the volatile compound levels and E-tongue sensor responses. Colors represent correlation coefficients, with red indicating a positive correlation and blue indicating a negative correlation. *, ** and *** represent significance at *p* < 0.05, *p* < 0.01 and *p* < 0.001, respectively.

The combined use of E-nose and HS-GC-IMS technologies enables effective discrimination of different LTB samples. The E-nose provides a comprehensive overview of the overall odor profile, while HS-GC-IMS enables precise characterization of treatment-specific VOCs. As shown in Figure 8a, cluster analysis classified the E-nose sensors into three distinct groups. The first group comprised W1S, W2S, W5S, and W6S sensors, which showed strong positive correlations with most VOCs and showed higher response intensities. The second group included W3S, W1W, and W2W sensors, exhibiting strong negative correlations with the majority of VOCs. The third group consisted of W3C, W1C, and W5C sensors, which demonstrated weaker negative correlations with most VOCs and relatively low response intensities. Detailed correlation analysis demonstrated that W1S, W2S, and W6S sensors were strongly positively correlated with butanal-M/D, 1-nonanal, pyrrolidine, 2-hexanol, 1-butanol,3-methyl-,acetate-M, 2-ethoxyethanol, benzene,propyl-M, 2,4,5-trimethylthiazole, ethyl heptanoate and trimethylamine compounds that were detected at higher concentrations in NL by HS-GC-IMS, which corroborated the corresponding higher response intensities of these E-nose sensors to NL. Meanwhile, W3S and W1W sensors, which showed higher responses to AL samples, were also positively correlated (though with weaker correlation coefficients compared to W1S and W2S) with benzene,propyl-M, 2-hexanol, pyrrolidine, butanal-D, and 1-nonanal. HS-GC-IMS analysis confirmed these compounds were present at significantly lower levels in AL than in NL, consistent with the correlation trends. Notably, potential chemical markers for GI-AL, including THF-M/D, 1,1-diethoxy ethane-D, and cyclohexane, showed positive correlations with the W3C sensor, aligning well with both their elevated concentrations in GI-AL detected by HS-GC-IMS and the stronger W3C sensor responses to GI-AL.

Figure 8b reveals significant correlations between E-tongue sensor responses and flavor compounds. While CTS and PKS sensors showed negative correlations with most VOCs, other sensors generally exhibited positive correlations. Notably, potential GI-AL markers (THF-M/D, 1,1-diethoxy ethane-D, and cyclohexane) demonstrated strong positive correlations with CTS sensors, consistent with E-tongue results showing enhanced saltiness in GI-AL compared to NL and 0 kGy-AL. Furthermore, CPS, AHS, and NMS sensors all showed significant positive correlations with compounds including 2-methyl butanoic acid ethyl ester, butanoic acid propyl ester, 2-butanone-M, 1-propanol, triethylamine, and propanal-M/D, indicating these compounds contribute to sour and umami tastes. Similarly, the significant positive correlations of SCS and ANS sensors with 2-methyl butanal-M/D, 1-hexanol, 2-ethoxyethanol, 1,1-diethoxy ethane-M, and ethyl heptanoate demonstrate their contributions to sweet and bitter tastes.

## 4. Conclusions

This study aims to systematically investigate the effects of GI treatment prior to aging on VOCs in LTB by integrating the analytical strengths of E-nose, E-tongue, and HS-GC–IMS—specifically, the rapid global flavor profiling capability of E-nose and E-tongue, coupled with the high-resolution molecular characterization offered by HS-GC–IMS. HS-GC-IMS analysis identified 30 characteristic VOCs, revealing that compared to NL, AL showed significantly increased ester content and decreased alcohol levels, while GI-AL exhibited marked increases in furans and alkanes relative to 0 kGy-AL. Notably, THF-M/D, 1,1-diethoxy ethane-D, and cyclohexane exhibited substantially higher concentrations in GI-AL compared to both NL and 0 kGy-AL, demonstrating distinct dose-dependent accumulation patterns. PLS-DA confirmed these compounds had VIP scores >1, establishing their strong potential as molecular markers for identifying GI-treated LTB. Correlation analyses between sensor responses (E-nose/E-tongue) and HS-GC-IMS VOC data confirmed the consistency between these complementary detection techniques. Beyond elucidating the significant flavor differences induced by GI treatment in AL, this study established integrated flavor evaluation models combining “E-nose & HS-GC-IMS” and “E-tongue & HS-GC-IMS”. These models provide a novel technological approach for both rapid flavor evaluation of light-flavor Baijiu and identification of GI treatment. Depending on the sensory focus, researchers can combine HS-GC-IMS with either the E-nose for aroma-driven studies or the E-tongue for taste-oriented analysis, enabling targeted sample discrimination. It should be noted that the application of GI in Baijiu production remains exploratory, requiring a careful balance between preserving traditional techniques and adopting technological innovation. For distinctive varieties such as LTB, its practical potential depends on identifying suitable irradiation-dose windows and treatment protocols that harmonize maturation control, flavor modulation, and product stability—a key direction for our subsequent research.

## Figures and Tables

**Table 1:** Performance description of E-nose sensors.

Sensors	Responsive Substance	Performance Description
W1C	aromatic	Sensitive to aromatic compounds
W5S	broad range	Sensitive to nitrogen oxides
W3C	aromatic	Sensitive to ammonia, aromatic compounds
W6S	hydrogen	Sensitive to hydrides
W5C	arom-aliph	Sensitive to short-chain alkanes, aromatic compounds
W1S	broad-methane	Sensitive to methyl
W1W	sulfur-organic	Sensitive to sulfides, pyrazine, many terpenes
W2S	broad-alcohol	Sensitive to alcohols, aldehydes and ketones
W2W	sulf-chlor	Sensitive to organic sulfides, aromatic compounds
W3S	methane-aliph	Sensitive to long-chain alkanes

**Table 2:** Information on the volatile organic compounds in LTB samples identified via GC-IMS.

No.	Compound	CAS	Molecule Formula	RI ^1^	RT ^2^	DT ^3^	Odor Description	Peak Intensities					
								NL	AL	2 kGy-AL	4 kGy-AL	6 kGy-AL	8 kGy-AL
Esters	butanoic acid propyl ester	105-66-8	C_7_H_14_O_2_	1144.9	575.844	1.2601	Fruity, sweet, apricot, pineapple	923.878 ± 8.096 ^a^	188.956 ± 10.588 ^cd^	177.799 ± 8.471 ^d^	204.226 ± 19.261 ^bc^	211.072 ± 3.733 ^b^	216.697 ± 7.293 ^b^
	acetic acid hexyl ester	142-92-7	C_8_H_16_O_2_	1284.2	983.138	1.38484	Fruity, green, apple, banana, sweet	1027.111 ± 9.525 ^a^	207.495 ± 14.308 ^b^	204.152 ± 9.471 ^bc^	185.885 ± 14.210 ^cd^	177.250 ± 6.210 ^d^	191.361 ± 5.463 ^bcd^
	ethyl (E)-2-butenoate	623-70-1	C_6_H_10_O_2_	1144.9	575.844	1.18408	Pungent, chemical, diffusive, sweet	195.727 ± 8.338 ^a^	110.043 ± 7.798 ^bc^	106.031 ± 7.160 ^d^	117.803 ± 3.058 ^bc^	114.596 ± 3.839 ^bc^	122.501 ± 6.851 ^b^
	ethyl heptanoate	106-30-9	C_9_H_18_O_2_	1341.1	1156.615	1.41341	Fruity, pineapple, cognac, rum, wine	322.713 ± 20.413 ^a^	153.489 ± 10.604 ^bc^	154.451 ± 12.206 ^bc^	179.581 ± 14.502 ^b^	145.481 ± 3.327 ^d^	146.986 ± 12.912 ^d^
	acetic acid ethyl ester-M	141-78-6	C_4_H_8_O_2_	915.1	296.668	1.09468	Ethereal, fruity, sweet, weedy, green	703.637 ± 68.814 ^a^	750.250 ± 13.162 ^a^	797.412 ± 36.243 ^a^	783.295 ± 29.878 ^a^	758.996 ± 60.956 ^a^	772.942 ± 67.769 ^a^
	acetic acid ethyl ester-D			905	290.9	1.33529		13,485.390 ± 174.424 ^b^	19,744.185 ± 58.267 ^a^	19,324.582 ± 364.901 ^a^	19,644.738 ± 262.318 ^a^	19,291.367 ± 438.157 ^a^	19,272.821 ± 301.391 ^a^
	2-methyl butanoic acid ethyl ester	7452-79-1	C_7_H_14_O_2_	1053.1	412.867	1.64724	Sharp, sweet, green, apple, fruity	468.171 ± 9.212 ^a^	334.654 ± 11.322 ^cd^	325.738 ± 19.702 ^d^	365.098 ± 22.717 ^bc^	370.118 ± 15.313 ^b^	391.898 ± 13.407 ^b^
	methyl acetate	79-20-9	C_3_H_6_O_2_	863.9	267.278	1.03088	Ether, sweet, fruity	414.136 ± 2.724 ^c^	548.799 ± 14.344 ^a^	459.456 ± 4.863 ^b^	449.232 ± 6.462 ^b^	452.032 ± 7.612 ^b^	457.768 ± 9.654 ^b^
Aldehydes	1-nonanal	124-19-6	C_9_H_18_O	1400.9	1335.916	1.48015	Waxy, aldehydic, rose, fresh	424.981 ± 17.924^a^	364.105 ± 10.040^b^	298.900 ± 7.118^c^	348.935 ± 39.944^b^	275.564 ± 26.034^c^	266.411 ± 26.836^c^
	1-hexanal-M	66-25-1	C_6_H_12_O	1039.9	396.777	1.26289	Fresh, green, fatty, aldehydic	527.442 ± 2.441 ^b^	553.357 ± 5.584 ^a^	555.534 ± 8.894 ^a^	497.070 ± 19.214 ^c^	509.958 ± 10.210 ^bc^	530.163 ± 20.393 ^ab^
	1-hexanal-D			1039.7	396.551	1.56126		8200.791 ± 42.808 ^a^	1773.754 ± 38.883 ^cd^	1687.590 ± 50.659 ^d^	1813.594 ± 42.299 ^bc^	1814.443 ± 47.091 ^bc^	1884.910 ± 46.254 ^b^
	2-methyl butanal-M	96-17-3	C_5_H_10_O	919.4	299.14	1.15426	Musty, cocoa, phenolic, coffee, nutty	180.782 ± 9.868 ^a^	99.938 ± 3.380 ^b^	99.790 ± 1.340 ^b^	110.794 ± 2.938 ^b^	107.241 ± 10.164 ^b^	98.596 ± 7.709 ^b^
	2-methyl butanal-D			922.8	301.117	1.391		525.614 ± 57.335 ^a^	148.271 ± 1.752 ^b^	145.200 ± 11.906 ^b^	133.698 ± 2.744 ^b^	152.234 ± 23.635 ^b^	165.444 ± 30.603 ^b^
	2,6-dimethyl-5-heptenal	106-72-9	C_9_H_16_O	1355.4	1199.448	1.30217	Fresh, ozone, melon	5524.674 ± 191.387 ^a^	2245.362 ± 61.007 ^b^	2223.470 ± 76.847 ^b^	2261.815 ± 81.167 ^b^	2275.898 ± 84.389 ^b^	2187.763 ± 129.830 ^b^
	butanal-M	123-72-8	C_4_H_8_O	854	261.566	1.11325	Pungent, cocoa, musty, green, malty	1876.854 ± 8.491 ^a^	1079.000 ± 14.055 ^b^	1052.407 ± 9.922 ^c^	1021.478 ± 12.753 ^d^	1009.623 ± 12.567 ^d^	1026.537 ± 9.859 ^d^
	butanal-D			849.7	259.094	1.28346		1847.846 ± 14.648 ^a^	1114.890 ± 13.679 ^b^	1021.191 ± 16.966 ^cd^	1034.554 ± 7.364 ^c^	1003.490 ± 10.032 ^d^	971.688 ± 4.129 ^e^
	propanal-M	123-38-6	C_3_H_6_O	840.5	253.82	1.06296	Earthy, alcohol, wine, whiskey, cocoa	873.231 ± 3.168 ^a^	438.923 ± 13.293 ^e^	439.206 ± 9.255 ^e^	474.391 ± 11.917 ^d^	512.589 ± 9.742 ^c^	547.150 ± 10.128 ^b^
	propanal-D			839.1	252.996	1.14961		733.704 ± 15.186 ^a^	158.417 ± 14.038 ^d^	172.749 ± 4.877 ^d^	205.467 ± 7.947 ^c^	221.111 ± 7.652 ^c^	260.087 ± 5.172 ^b^
	1,1-diethoxy ethane-M	105-57-7	C_6_H_14_O_2_	882.6	278.016	1.04105	Ether, green, nut, earthy, sweet	1051.131 ± 2.873 ^a^	963.739 ± 101.801 ^ab^	1021.800 ± 8.078 ^a^	1021.943 ± 18.267 ^a^	970.425 ± 16.598 ^ab^	909.057 ± 13.027 ^b^
	1,1-diethoxy ethane-D			877.2	274.908	1.12854		592.010 ± 2.475 ^e^	528.169 ± 16.228 ^f^	1377.047 ± 11.811 ^d^	1568.033 ± 15.001 ^c^	1742.352 ± 6.627 ^b^	1896.054 ± 17.372 ^a^
Ketones	2,6-dimethyl-4-heptanone (diisobutyl ketone)-D	108-83-8	C_9_H_18_O	1213.1	751.935	1.78466	Green, fruity, metallic, pineapple	2520.953 ± 54.303 ^a^	705.456 ± 10.253 ^b^	666.913 ± 7.329b ^c^	678.440 ± 3.249b ^c^	648.526 ± 8.805 ^c^	672.823 ± 37.609 ^bc^
	2,6-dimethyl-4-heptanone (diisobutyl ketone)-M			1219.7	773.238	1.32197		1649.115 ± 17.497 ^a^	1401.751 ± 21.146 ^bc^	1399.497 ± 25.086 ^bc^	1363.228 ± 30.525 ^c^	1396.375 ± 11.621 ^bc^	1444.641 ± 44.076 ^b^
	1-penten-3-one	1629-58-9	C_5_H_8_O	1045.7	403.862	1.31242	Pungent, peppery, mustard, garlic	10,348.930 ± 34.658 ^a^	7313.119 ± 131.528 ^b^	7126.886 ± 81.119 ^c^	7307.276 ± 55.942 ^b^	7322.450 ± 70.090 ^b^	7350.268 ± 26.393 ^b^
	2-butanone-M	78-93-3	C_4_H_8_O	913	295.514	1.05755	acetone-like, ethereal, fruity, camphor	249.153 ± 30.569 ^a^	30.488 ± 1.128 ^b^	33.523 ± 5.506 ^b^	32.231 ± 2.598 ^b^	36.055 ± 5.746 ^b^	42.494 ± 6.035 ^b^
	2-butanone-D			911.9	294.855	1.23859		464.573 ± 44.840 ^a^	55.350 ± 1.805 ^b^	60.200 ± 4.679 ^b^	50.479 ± 0.788 ^b^	53.339 ± 3.031 ^b^	58.654 ± 4.000 ^b^
Alcohols	2-ethoxyethan-ol	110-80-5	C_4_H_10_O_2_	1240.5	840.907	1.34095	Sweet, chemical, solvent	8098.330 ± 145.490 ^a^	1812.018 ± 24.400 ^b^	1802.096 ± 148.985 ^b^	1896.647 ± 33.848 ^b^	1737.320 ± 134.318 ^b^	1884.977 ± 13.822 ^b^
	1-butanol, 3-methyl-, acetate-M	123-92-2	C_7_H_14_O_2_	1132.3	547.083	1.31035	Sweet, fruity, banana, solvent	5929.7110 ± 37.17524 ^a^	4857.467 ± 87.897 ^b^	4728.180 ± 148.227 ^b^	4441.476 ± 90.696 ^c^	4362.284 ± 97.539 ^c^	4521.469 ± 15.999 ^c^
	1-butanol, 3-methyl-, acetate-D			1128.4	537.964	1.74585		30,958.243 ± 303.796 ^a^	10,475.319 ± 201.244 ^b^	9586.736 ± 330.154 ^d^	9940.523 ± 119.759 ^cd^	9688.442 ± 187.149 ^d^	10,158.220 ± 146.655 ^bc^
	1-propanol, 2-methyl-D	78-83-1	C_4_H_10_O	1099.8	472.726	1.37477	Ethereal, winey, cortex	17,022.254 ± 215.907 ^a^	7147.402 ± 79.111 ^b^	6885.715 ± 244.119 ^b^	7140.749 ± 154.347 ^b^	7000.405 ± 220.909 ^b^	7199.540 ± 16.221 ^b^
	1-propanol, 2-methyl-M			1101.6	476.935	1.16476		1494.303 ± 8.934 ^a^	1489.829 ± 6.813 ^a^	1489.548 ± 12.700 ^a^	1451.708 ± 10.341 ^a^	1492.153 ± 45.583 ^a^	1449.588 ± 67.611 ^a^
	1-propanol	71-23-8	C_3_H_8_O	1054	413.991	1.24941	Alcoholic, fermented, fusel, musty	1082.895 ± 10.975 ^a^	826.379 ± 28.386 ^c^	860.538 ± 49.474 ^bc^	902.836 ± 40.026 ^bc^	913.865 ± 65.337 ^b^	932.052 ± 34.434 ^b^
	1-hexanol	111-27-3	C_6_H_14_O	1364.7	1227.339	1.32784	Ethereal, fusel, oil, fruity, alcoholic, sweet	574.193 ± 19.274 ^a^	119.210 ± 4.685 ^b^	123.171 ± 7.897 ^b^	132.943 ± 10.724 ^b^	133.159 ± 24.424 ^b^	121.061 ± 4.746 ^b^
	2-hexanol	626-93-7	C_6_H_14_O	1289	998.802	1.28281	Chemical, winey, fruity, fatty, terpenic	382.455 ± 11.182 ^a^	171.504 ± 9.613 ^b^	149.614 ± 10.884 ^b^	120.948 ± 6.335 ^c^	108.187 ± 8.986 ^c^	107.566 ± 14.369 ^c^
Furans	tetrahydrofur-an	109-99-9	C_4_H_8_O	859.4	264.697	1.06683	Chemical, sweet, ether-like	533.722 ± 2.524 ^e^	664.430 ± 6.973 ^d^	707.757 ± 8.568 ^a^	708.468 ± 12.068 ^a^	693.393 ± 0.244b ^c^	685.253 ± 20.911 ^cd^
	tetrahydrofur-an-M			788.5	223.914	1.0636		199.590 ± 12.041 ^e^	277.963 ± 11.443 ^d^	553.414 ± 10.829 ^c^	646.072 ± 30.157 ^b^	722.144 ± 6.876 ^a^	736.253 ± 2.088 ^a^
	tetrahydrofur-an-D			788.5	223.914	1.22865		13.644 ± 0.894 ^e^	39.892 ± 2.843 ^e^	288.428 ± 8.771 ^d^	466.107 ± 47.111 ^c^	720.564 ± 24.386 ^b^	935.744 ± 51.134 ^a^
Alkanes	cyclohexane	110-82-7	C_6_H_12_	781.8	220.075	1.12944	-	143.054 ± 1.385 ^e^	118.650 ± 8.458 ^e^	1025.335 ± 22.891 ^d^	1460.442 ± 127.862 ^c^	2011.234 ± 56.689 ^b^	2380.843 ± 94.797 ^a^
	pyrrolidine	123-75-1	C_4_H_9_N	989.5	339.426	1.27501	Ammoniacal, animal, egg, amine	1439.644 ± 22.834 ^a^	1047.054 ± 12.997 ^b^	1027.201 ± 13.950 ^bc^	1029.867 ± 5.482 ^bc^	1023.656 ± 10.987 ^bc^	1017.161 ± 6.350 ^c^
Thiazoles	2,4,5-trimethyl-thiazole	13623-11-5	C_6_H_9_NS	1401.6	1337.908	1.56743	Musty, nutty, vegetable, cocoa, hazelnut	424.594 ± 15.524 ^a^	335.020 ± 10.637 ^c^	319.343 ± 3.123 ^c^	368.671 ± 18.146 ^b^	308.727 ± 6.340 ^c^	312.460 ± 26.433 ^c^
Others	benzene, propyl-M	103-65-1	C_9_H_12_	1218.3	768.852	1.25197	-	34,037.599 ± 164.753 ^a^	12,537.683 ± 126.410 ^b^	12,012.908 ± 14.986 ^c^	12,357.092 ± 107.475 ^b^	12,403.730 ± 233.682 ^b^	12,424.519 ± 88.692 ^b^
	benzene, propyl-D			1213.3	752.561	1.49992		7451.671 ± 45.964 ^a^	5413.559 ± 107.641 ^b^	5221.222 ± 44.866 ^c^	4979.499 ± 74.026 ^d^	4910.556 ± 37.855 ^d^	4930.134 ± 23.591 ^d^
	1,4-dioxan-M	123-91-1	C_4_H_8_O_2_	1031	385.888	1.13806	-	4468.057 ± 17.619 ^c^	7733.569 ± 80.337 ^ab^	7756.122 ± 33.418 ^ab^	7732.069 ± 53.338 ^ab^	7838.149 ± 100.203 ^a^	7665.125 ± 17.462 ^b^
	1,4-dioxan-D			1027.2	381.258	1.33221	-	6222.433 ± 42.054 ^a^	3062.638 ± 8.459 ^bc^	2930.951 ± 51.470 ^d^	3006.338 ± 60.314 ^cd^	3040.349 ± 30.157 ^bc^	3125.018 ± 41.568 ^b^
	triethylamine	121-44-8	C_6_H_15_N	823.6	244.097	1.09082	Ammoniacal, fishy	544.933 ± 45.982 ^a^	124.395 ± 5.505 ^c^	161.523 ± 6.895b ^c^	182.660 ± 4.052 ^b^	174.362 ± 5.800 ^b^	186.103 ± 3.329 ^b^

Odor descriptions are obtained from the Flavornet database (http://www.perflavory.com/search.php, accessed on 10 May 2025; http://thegoodscentscompany.com, accessed on 10 May 2025). The peak intensity values are the means ± standard deviation. Values in the same line with different letters (a–f) are significantly different (*p* < 0.05); - represents not found; ^1^ represents the retention indexes of the volatile compounds in the GC column. ^2^ represents the retention time in the capillary GC column; ^3^ represents the drift time in the drift tube.

## Data Availability

The data presented in this study are available on request from the corresponding author. At present, China has no specific irradiation standards or legal regulations specifically for Baijiu products.

## Abbreviations

The following abbreviations are used in this manuscript:

GI	Gamma irradiation
LTB	Light-flavor tartary buckwheat Baijiu
HS-GC-IMS	Headspace–gas chromatography–ion mobility spectrometry
VOCs	Volatile organic compounds
NL	Newly brewed LTB
0 kGy-AL	Naturally aged 3-year LTB
GI-AL	Gamma-irradiated then aged 3-year LTB
AL	AL is a collective term for all LTB that have undergone the aging process, encompassing both 0kGy-AL and GI-AL
2/4/6/8 kGy-AL	Gamma-irradiated (various doses) then aged 3-year LTB
